# Intrathecal nicardipine for symptomatic, refractory vasospasm treatment in pediatric patients: a case series

**DOI:** 10.1007/s00381-025-06868-4

**Published:** 2025-06-09

**Authors:** Lucinda Chiu, Jillian Plonsker, Nicole Dayanara Villalba, Michael DeCuypere, Tord Alden, Jonathan Scoville, Sandi Lam

**Affiliations:** 1 Division of Pediatric Neurosurgery, Department of Neurosurgery, Ann and Robert H. Lurie Children’s Hospital of Chicago, Northwestern University Feinberg School of Medicine, Chicago, IL USA; 2 Department of Neurological Surgery, Rush University Medical Center, Chicago, IL USA

**Keywords:** Vasospasm, Intrathecal nicardipine, Non-traumatic subarachnoid hemorrhage, Delayed cerebral ischemia, Neurocritical care, Pediatric

## Abstract

**Purpose:**

Vasospasm is a potentially devastating condition that can develop in pediatric patients secondary to traumatic, inflammatory, infectious, and neoplastic etiologies and carries a high risk of morbidity and mortality (Isola et al. 2022, Krishna et al. 2005). Management is often adapted from adult to pediatric care given its relative rarity in children. There is level 3 evidence for the use of intrathecal (IT) calcium channel blocker (nicardipine) administration to treat refractory vasospasm in the adult subarachnoid hemorrhage (SAH) population. Its use has yet to be investigated in children (Krishna et al. 2005). We aim to review the safety profile and efficacy of IT nicardipine for non-traumatic SAH with refractory vasospasm at our institution.

**Methods:**

We present a single institution retrospective series of pediatric patients treated with IT nicardipine for symptomatic vasospasm refractory to standard therapies including oral nimodipine, hyperdynamic augmentation therapies, and targeted intra-arterial therapy.

**Results:**

Pediatric patients (*n* = 3), ages 16–17 years old, admitted with non-traumatic SAH who developed severe, symptomatic, and refractory vasospasm were included. All patients presented with SAH from ruptured aneurysms. A 5-day course of IT nicardipine was initiated using a protocol adapted from our adult cerebrovascular/neurocritical care service. Decreases in peak middle cerebral artery velocity (range 61–290 cm/s) and Lindegaard ratios (range 0.3–4.2) were observed from initiation to completion of treatment.

**Conclusions:**

IT nicardipine for pediatric non-traumatic SAH with vasospasm refractory to standard treatments was used in our small series with no complications and no new ischemic events. Clinical and radiographic effects were encouraging. Further study is warranted.

## Introduction

Cerebral vasospasm is a potentially devastating neurological condition that carries a high risk of morbidity and mortality [1]. Vasospasm is characterized by narrowing of the intracranial blood vessel lumen and can occur secondary to traumatic, inflammatory, infectious, and neoplastic etiologies. Cerebral vasospasm in pediatric patients is a common complication of subarachnoid hemorrhage (SAH), with an incidence of 21 to 57% in non-traumatic SAH cases [1–3]. Intrathecal (IT) administration of nicardipine has been shown to be safe and effective in adults with symptomatic, refractory vasospasm, but its role in pediatric cerebral vasospasm (PCV) has not been well studied [4–6]. Here, we present three cases of IT nicardipine use to illustrate its safety and potential efficacy for treating symptomatic, refractory PCV secondary to SAH.

## Historical background

John Hunter first described arterial vessel contraction in 1837. Vasospasm was described in 1951 after angiograms following aneurysm rupture showed reversible intradural arterial narrowing [7]. Transcranial Doppler (TCD) was introduced by Rune Aaslid in the early 1980 s [8]. Studies discussing PCV secondary to SAH increased after a 2005 study compared clinicoradiographic and surgical outcomes of adult versus pediatric intracranial aneurysms [2]. Nimodipine, a calcium channel blocker (CCB), was approved by the US Food and Drug Administration in 1988. In the 1990 s, IT nicardipine, another CCB which presumably acts through its vasodilatory effect of distal cerebral vessels, was reported to have promising clinical benefits for symptomatic vasospasm [9–13].

## Clinical presentation and diagnosis

Patients typically present with PCV within a few days to 1 week following their initial bleed. Presenting symptoms vary with the affected vessel and can present gradually or precipitously [14].

In addition to serial neurological examinations, radiographic monitoring modalities are used for vasospasm diagnosis. Digital subtraction angiography (DSA) is the gold standard for detecting PCV and allows for simultaneous diagnosis and treatment, but is an invasive procedure [15]. TCD ultrasonography is noninvasive and can be done bedside, but is operator dependent and has lower sensitivity [16]. Computed tomography angiography (CTA) and magnetic resonance angiography (MRA) have high specificity for vasospasm, but depend upon equipment accessibility and typically require patient transport [1, 17].

PCV secondary to non-traumatic SAH remains a rare pathology with most centers managing one to three aneurysmal SAH cases annually [18]. Many pediatric SAH protocols are based on adaptations of adult guidelines. While some studies have shown potential benefits of extrapolating from the adult SAH population to the pediatric one, others have shown limited utility for PCV surveillance secondary to practice variations and lack of standardized normative pediatric values [19, 20]. For example, TCD is commonly used for adult vasospasm surveillance with standard thresholds for vasospasm diagnosis [21–23]. In the pediatric population, TCD use is not universal; values followed for PCV diagnosis also vary [18]. Prior studies suggest that adult TCD criteria may overestimate the true prevalence of PCV, especially that of symptomatic PCV, given higher baseline flow velocities in children than adults [20, 24]. No LR value has been validated in children for vasospasm diagnosis; thus, a recent Delphi consensus statement of TCD use in the pediatric ICU (PICU) setting found that trends in measured velocities are preferred [25].

## Management, prognosis, and outcomes

Given the risk for permanent neurologic deficits secondary to hypoperfusion from vasospasm, the goal of care for SAH patients is to prevent delayed cerebral ischemia (DCI). Without prompt recognition and proper treatment, DCI from PCV is associated with increased morbidity and mortality [26, 27].

Medical management strategies for symptomatic cerebral vasospasm include oral nimodipine, maintenance of euvolemia, and targeted hypertensive therapy [5]. In cases where symptomatic vasospasm is refractory to medical therapy, endovascular strategies, such as intra-arterial (IA) vasodilators and balloon angioplasty, can be used to alleviate vasospasm and improve cerebral blood flow [28, 29].

IT administration of nicardipine has been shown to be safe and effective in adults and is incorporated into treatment protocols for cerebral vasospasm at some institutions [11, 30, 31]. They report this approach may help patients avoid systemic side effects, such as hypotension, which is commonly associated with oral and IA calcium channel blockers, and reduce the need for invasive endovascular interventions [32].

## Exemplary case descriptions

This single institution, retrospective case series includes children ≤ 18 years who were admitted to our hospital’s PICU in 2023 with non-traumatic SAH and who completed a 5-day course of IT nicardipine. IT nicardipine was discussed in a multidisciplinary fashion prior to initiation for patients who developed severe, symptomatic vasospasm, diagnosed clinically and/or radiographically, that was refractory to standard clinical therapies, including oral nimodipine, hyperdynamic augmentation, and euvolemia. Each patient received 4 mg of nicardipine every 8 h through their existing EVD, which was placed on admission for hydrocephalus secondary to their SAH. The EVD access hub was sanitized by a provider and clamped distally. Two milliliters (mL) of CSF was aspirated. Two mL of nicardipine (concentration, 2.5 mg/mL solution) was injected proximally, followed with 5 mL of 0.9% sodium chloride solution, in order to minimize the amount of nicardipine in the EVD tubing. The EVD was clamped for 1 h. Intracranial pressures (ICP) were monitored throughout the patient’s admission. Vessel imaging was obtained upon completion of the IT nicardipine treatment. This protocol was adapted from one used by our adult neurocritical care service.

**Fig. 1:**
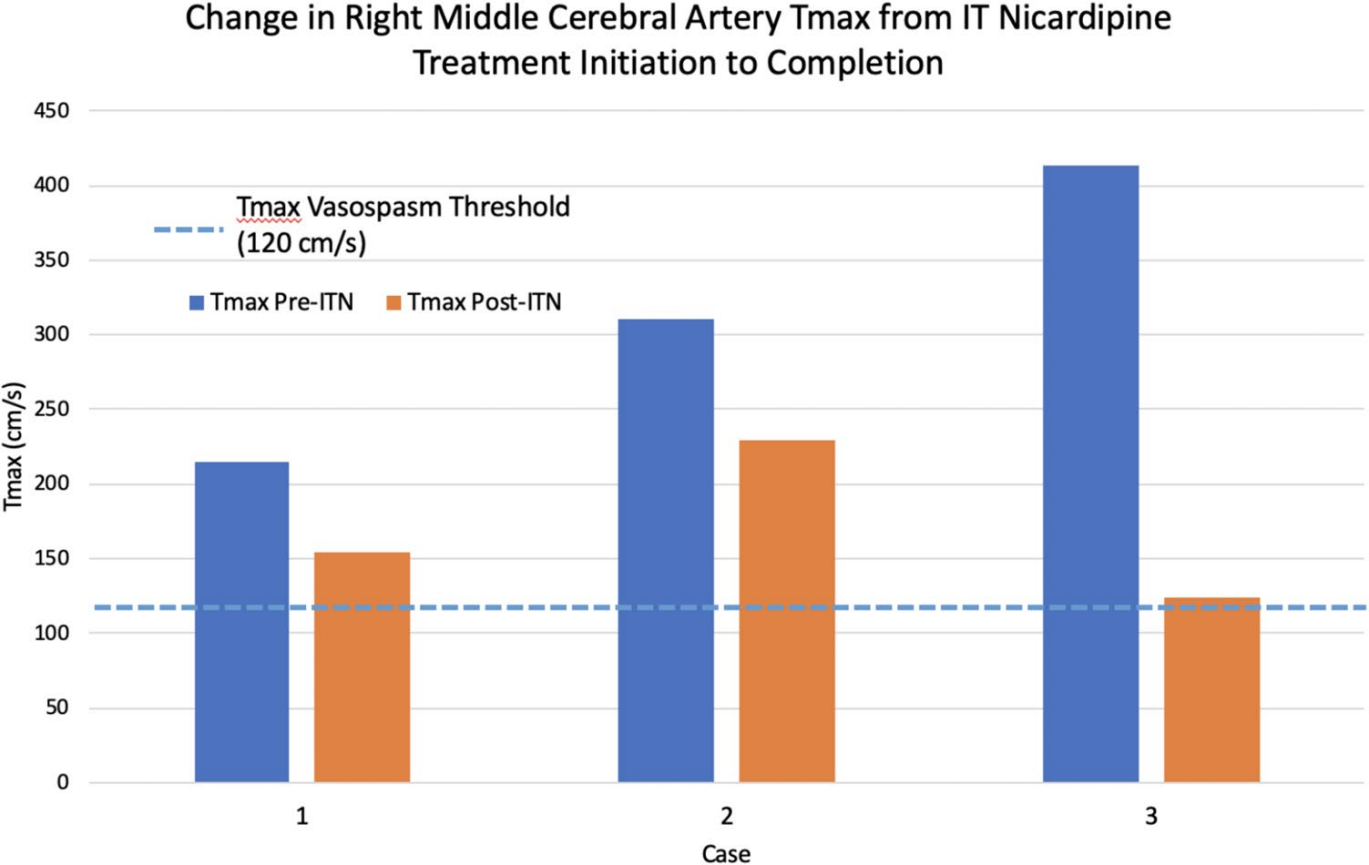
Bar graph illustrating differences in transcranial Doppler (TCD) right proximal middle cerebral artery (MCA) peak systolic velocities (Tmax, cm/s) for all three patients from immediately pre-intrathecal nicardipine (ITN) administration to immediately post-ITN administration. Tmax pre-ITN is represented in *blue*. Tmax post-ITN is represented in *orange*. The standard adult vasospasm threshold is represented with a *dashed line* at 120 cm/s. All three patients had a decrease in MCA Tmax from initiation to completion of ITN treatment (mean − 144 cm/s, median − 82 cm/s). Case 3 had the greatest difference in MCA Tmax of − 290 cm/s (414 cm/s pre-ITN to 124 cm/s post-ITN)

Three patients met inclusion criteria. The median age was 17 years. One patient was female. Hunt and Hess (HH) score on presentation varied from two to four. All patients presented with a modified Fisher scale (mFS) score of 4. Two patients presented with SAH secondary to a ruptured anterior communicating artery (AcoA) aneurysm. The third presented with a ruptured dissecting pseudoaneurysm. IT nicardipine was initiated on PBD 9 in two patients and on PBD 3 in the other patient. No patients had elevated ICP (> 20 cm of water) following administration of IT nicardipine or during EVD clamping. MCA Tmax and LR decreased for all patients from day 1 to day 5 of IT nicardipine (Fig. 1). Average length of stay was 30 days. Follow-up ranged from 9 to 17 months (Table 1).

**Table 1:** Patient demographics

Patient	Age (years)	Sex	HH	mFS	SAH etiology	IT nicardipine initiation (PBD)	MCA peak velocity (Tmax) *∆* (cm/s)	Lindegaard ratio *∆*	Length of follow-up (months)
1	16	F	4	4	Ruptured AcoA	3	− 61	− 0.3	12
2	17	M	2	4	Ruptured AcoA	9	− 82	− 4.2	9
3	17	M	3	4	Ruptured right A2 pseudoaneurysm	9	− 290	− 1.1	17

*∆* is calculated as the difference between the Tmax or Lindegaard ratio value closest to the first and last day of IT nicardipine therapy

*HH* Hunt and Hess, *mFS* modified Fischer scale, *SAH* subarachnoid hemorrhage, *PBD* post-bleed day, *MCA* middle cerebral artery, *Tmax (cm/s)* peak systolic velocity (centimeters/second)

### Case 1

A 16-year-old female presented as a HH4, mF4 from a ruptured right ACoA aneurysm. The patient was intubated, and a left frontal EVD was placed. The patient underwent successful coil embolization without residual filling. SBP augmentation was initiated and nimodipine administered; three vasopressors were required to achieve SBP goals.

**Fig. 2:**
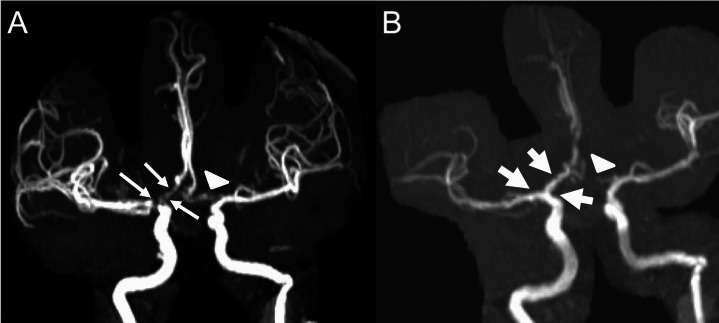
Vessel and cranial imaging for Case 1. **A** Magnetic resonance angiography (MRA) anterior circulation reconstructions obtained on PBD 3 prior to IT nicardipine initiation showing new narrowing of the right terminal internal carotid artery (ICA), M1, and A1 origins, concerning for vasospasm (thin arrows). A likely developmentally hypoplastic left A1 segment is noted (arrowhead). **B** MRA anterior circulation reconstructions obtained on PBD 9 following completion of IT nicardipine therapy with improved right ICA, M1, and A1 caliber compared to prior imaging (thick arrows). The developmentally hypoplastic left A1 is again seen (arrowhead)

On PBD 2–3, the patient was febrile and had decreased movement of her right side, concerning for vasospasm. All fever workup remained negative. Magnetic resonance imaging (MRI) brain stroke protocol with MRA showed diffusion restriction of bilateral frontal and parietal lobes and new narrowing of the right terminal internal carotid artery (ICA), A1, and M1 segments (Fig. 2A). TCD showed persistent elevation of the right LR at 3.7 and elevated right MCA Tmax (Fig. 1).

The development of vasospasm on PBD 2 raised concern that the patient’s urgent care visit for headache 2 weeks earlier was from a sentinel bleed. The patient underwent a DSA and received IA verapamil to the bilateral ICA. Residual distal right A1 narrowing was noted; an angioplasty was performed. Given the rapid radiographic progression of the patient’s strokes secondary to severe vasospasm without a reliable neurologic exam, IT nicardipine was initiated on PBD 3.

The patient’s clinical status and TCD subsequently improved. The patient completed IT nicardipine on PBD 8. MRI stroke with MRA on PBD 9 showed no acute ischemia and greatly improved right A1 and M1 caliber (Fig. 2B). The patient followed commands with 1/5 left-sided weakness. Care was de-escalated with extubation, discontinuation of TCD, and EVD removal. She was discharged to acute rehabilitation facility for intensive therapies.

Two months later, the patient was ambulating with a left ankle–foot orthosis. At 1-year follow-up, the patient attended school full time; her left side had nearly full motor strength (4 +/5 lower, 5/5 upper) except for a persistent foot drop. There were no new strokes, vasospasms, or aneurysm recurrences on the MRI/MRA.

### Case 2

A 17-year-old male presented as a HH2, mF4 from a ruptured 2 mm inferior-pointing AcoA aneurysm. A right frontal EVD was placed.

Surgical clipping was favored given the aneurysm’s small size and configuration and the patient’s young age. On PBD 3, the patient underwent a left craniotomy for aneurysm clipping. Direct visualization and intra-operative indocyanine green (ICG) runs showed complete occlusion of the aneurysm.

Despite standard therapies, left MCA velocities and LR began to uptrend. The patient was non-focal and experienced significant delirium and fevers with no positive cultures on fever workup.

On PBD 9, given persistently elevated left MCA velocities and LR, a 5-day course of IT nicardipine was initiated following multidisciplinary discussion. The patient experienced improvement in his mental status. TCD showed an overall decrease in Tmax and LR (Fig. 1). After the completion of the IT nicardipine course, CTA had no vasospasm; the left MCA had some irregularity but no significant narrowing. The patient’s EVD was removed and the patient was discharged to outpatient day rehabilitation.

At 9-month follow-up, he was attending university full time with no neurologic deficits. CT head/CTA had no residual aneurysm or new findings.

### Case 3

A 17-year-old male was admitted as a HH3, mF4 after vessel injury of the proximal right A2 segment. The patient was intubated, and a right frontal EVD was placed. SBP augmentation was initiated; two vasopressors were required.

The patient was febrile on PBD 6; all workup remained negative. Despite standard therapies, MRA and DSA showed new bilateral ICA and M1 narrowing, concerning for vasospasm. IA verapamil was injected into bilateral ICA. The patient’s ruptured right A2 pseudoaneurysm was successfully coiled. A 5-day course of IT nicardipine was initiated on PBD 9.

A repeat DSA on PBD 12 showed overall improved vasospasm, but persistent ACA spasm, and two small left A2 pseudoaneurysms, which were treated with a pipeline embolization device (PED). MRI PBD 13 did not show new acute ischemia.

The patient was extubated and completed IT nicardipine on PBD 14. TCD showed interval decreased bilateral MCA velocities (Fig. 1). The EVD was removed, and the patient was discharged to inpatient rehabilitation.

At 2-week follow-up, the patient was ambulatory and conversant. At 17-month follow-up, he continues to do well and remains in therapies for some behavioral changes and mild left-sided weakness (4/5). Follow-up DSA/MRI/MRA imaging showed no new bleeds or ischemia.

## Discussion

This is the largest retrospective case series, to our knowledge, reporting on the use IT nicardipine in the under-18 population for the treatment of symptomatic, severe, refractory vasospasm secondary to non-traumatic SAH. While IT nicardipine has shown promising benefits in the adult literature for improving vasospasm, currently, in the pediatric population, there are only two case reports documenting its successful use for refractory vasospasm in a 12-year-old secondary to meningitis at our institution and an 11-year-old secondary to traumatic SAH [5, 6, 12, 13, 33–35]. There is currently no consensus or guideline on vasospasm management in pediatrics.

### Safety of IT nicardipine

In the literature, the most commonly reported complications associated with IT nicardipine were central nervous system (CNS) infections, i.e., meningitis and/or ventriculitis. Rates ranged from 0 to 6%, though some of these patients who underwent open microsurgical treatment may have increased the risk of CNS infection [6, 12, 35]. Contemporary studies found that infection rates following intraventricular medication administration are similar to the infection risk of having an EVD alone [36, 37]. All three patients in our cohort developed fevers, which is not unusual in the setting of SAH. Full infectious workup, including CSF, was evaluated, and none of our patients had positive lab, imaging, or clinical findings of meningitis and/or ventriculitis.

Vessel imaging was obtained prior to and following completion of IT nicardipine in all patients. At our institution, daily TCD and an initial DSA are part of our SAH protocol. The modality of follow-up vessel imaging was decided case by case. In our patients, no new acute symptomatic or radiographic strokes secondary to vasospasm were seen at completion of IT nicardipine treatment. No patients developed new neurologic deficits during or following IT nicardipine.

All IT nicardipine therapies were administered through our patients’ existing EVD. No patients required permanent CSF diversion, a favorable outcome similar to two previously published case reports [5, 6]. Rates of shunt-dependent hydrocephalus after pediatric non-traumatic SAH are not known; they have been reported at 13–18% in adults following aneurysmal SAH [35, 38, 39].

### Efficacy of IT nicardipine

This study was a retrospective analysis of a rare disease in the pediatric population without a matched control group. Causality cannot be concluded.

All patients showed an overall decrease in measured TCD peak MCA velocities and LR from initiation to the completion of IT nicardipine treatment (Fig. 1). The average decrease of peak MCA velocities was 144 cm/s from initiation to the completion of IT nicardipine (median, − 82 cm/s). LR decreased on average by 1.86 (median, − 1.1). While there are no standard normative TCD values in the pediatric population, the trend seen here is reassuring. This trend has also been reported in larger sample size studies in the adult population with decreases in peak MCA velocity of 30–40 cm/s following treatment [33, 34, 40]. All three patients in our series also showed stable to improved vessel caliber on repeat vessel imaging post- compared to pre-IT nicardipine treatment. One patient had some possible MCA irregularity on post-treatment vessel imaging, but no significant narrowing concerning for vasospasm. All of our patients received the same modality of vessel imaging (CTA or MRA) at the completion of IT nicardipine treatment as they did prior to treatment initiation, allowing for more accurate comparisons of vessel caliber.

At latest follow-up, all patients had significant recovery from their initial presentation. All patients returned to school without special accommodations. These findings are similar to those in adults where IT nicardipine has been found to be associated with improved functional outcomes and reduced DCI [36].

### Pediatric vasospasm diagnosis

Diagnosis of PCV using TCD remains challenging given no standardized Tmax and LR values, as well as inherent technical challenges. In our cases, many TCD values were unreliable due to poor windows. In Case 3, TCDs were discontinued due to difficult windows. At a later attempt in the same patient, suboptimal windows led to overestimated LR values.

In Case 1, following the completion of IT nicardipine, the LR and Tmax continued to fluctuate despite improving neurologic status. These experiences support the need for further study examining the accuracy and role of TCD for PCV surveillance.

### Pediatric SAH care pathways

SAH care pathways in the pediatric population include multiple elements. These interventions are not all benign, and there may be a role for IT nicardipine.

Nimodipine chemoprophylaxis is the standard of care to prevent DCI after SAH in adults, but may not always be tolerated due to its systemic effects leading to hypotension [41]. All three of our patients had prophylactic nimodipine administration. Cases 2 and 3 had their nimodipine dose and/or frequency decreased due to hypotension. A recent survey found that 85% of pediatric care physicians (neurosurgeons, neurologists, and intensivists) surveyed used nimodipine [18]. IT nicardipine allows for more targeted, concentrated delivery of the therapy, decreasing the systemic effects; the frequency of hypotension from IT nicardipine remains unknown.

SBP augmentation with vasopressors is currently used in conjunction with euvolemia for PCV prophylaxis and treatment in the PICU [42]. SBP management again varies across providers and institutions. An age-specific goal is most commonly used prior to securing the aneurysm, but following definitive aneurysm treatment, SBP management became more individualized with 65.5% using an induced hypertension goal for PCV and DCI prevention [18]. Vasopressor use can have adverse effects, most notably excessive vasoconstriction causing organ infarction or vasospasm causing extremity ischemia [43, 44]. In our series, Case 1 had an unsuccessful angiogram due to femoral artery vasospasm, which was attributed to the effect of systemic vasopressors.

DSA with IA therapies remains the gold standard for vasospasm diagnosis and an effective treatment option [11, 24]. Endovascular interventions are used by the majority of providers in a recent survey to manage PCV, with up to 81% using IA verapamil and 67% using angioplasty [18]. This treatment option is not without its limitations: it is invasive and requires interventional radiology staff and space availability and clinical stability for patient transport. Case 2 did not undergo a DSA for IA therapies as the patient’s neurologic exam and CTA showed clinically significant vasospasm. Instead, IT nicardipine was started, and the patient had subsequent improvement in his TCD, repeat vessel imaging, and exam.

This case series demonstrates promising results of IT nicardipine in carefully selected pediatric patients. However, the safety and efficacy of IT nicardipine first need to be established, as well as the determination of its optimal dosing concentration, frequency of administration, and duration of treatment in the pediatric population. Once this is determined, studies exploring the role of IT nicardipine in the standard care pathway of refractory PCV treatment, in conjunction or in comparison with IA therapies, are warranted.

## Conclusions

Our study illustrates the use of IT nicardipine therapy in the treatment of severe, refractory vasospasm secondary to non-traumatic SAH in adolescent patients. While limited by a small sample size and a single institution, retrospective study design, IT nicardipine for PCV was used with no complications or new ischemic events. Clinical and radiographic effects were encouraging; this may hold promise as an additional treatment modality in the neurocritical care toolbox for appropriately selected patients. Larger cohort studies and multicenter, prospective trials with control groups are needed to further elucidate and validate the efficacy of IT nicardipine.

## Data Availability

No datasets were generated or analysed during the current study.
